# Secreted Effectors Modulating Immune Responses to *Toxoplasma gondii*

**DOI:** 10.3390/life11090988

**Published:** 2021-09-20

**Authors:** Tadakimi Tomita, Rebekah B. Guevara, Lamisha M. Shah, Andrews Y. Afrifa, Louis M. Weiss

**Affiliations:** 1 Department of Pathology, Albert Einstein College of Medicine, Bronx, NY 10461, USA; tadakimi.tomita@einsteinmed.org (T.T.); rebekah.guevara@einsteinmed.org (R.B.G.); 2 Department of Biological Science, Lehman College of the City University of New York, Bronx, NY 10468, USA; lamisha.shah@lc.cuny.edu (L.M.S.); andrews.afrifa@lc.cuny.edu (A.Y.A.); 3 Department of Medicine, Albert Einstein College of Medicine, Bronx, NY 10461, USA

**Keywords:** *Toxoplasma gondii*, secreted effector, innate immunity, dense granule proteins, immune modulation

## Abstract

*Toxoplasma gondii* is an obligate intracellular parasite that chronically infects a third of humans. It can cause life-threatening encephalitis in immune-compromised individuals. Congenital infection also results in blindness and intellectual disabilities. In the intracellular milieu, parasites encounter various immunological effectors that have been shaped to limit parasite infection. Parasites not only have to suppress these anti-parasitic inflammatory responses but also ensure the host organism’s survival until their subsequent transmission. Recent advancements in *T. gondii* research have revealed a plethora of parasite-secreted proteins that suppress as well as activate immune responses. This mini-review will comprehensively examine each secreted immunomodulatory effector based on the location of their actions. The first section is focused on secreted effectors that localize to the parasitophorous vacuole membrane, the interface between the parasites and the host cytoplasm. Murine hosts are equipped with potent IFNγ-induced immune-related GTPases, and various parasite effectors subvert these to prevent parasite elimination. The second section examines several cytoplasmic and ER effectors, including a recently described function for matrix antigen 1 (MAG1) as a secreted effector. The third section covers the repertoire of nuclear effectors that hijack transcription factors and epigenetic repressors that alter gene expression. The last section focuses on the translocation of dense-granule effectors and effectors in the setting of *T. gondii* tissue cysts (the bradyzoite parasitophorous vacuole).

## 1. Introduction

*Toxoplasma gondii* is a prevalent pathogen that can infect any warm-blooded animal, including humans. It can cause severe encephalitis in immune-compromised individuals such as patients with AIDS or transplant recipients. Infection of a serologically naïve mother with *T. gondii* can give rise to congenital infection of the fetus resulting in miscarriage, intellectual disability, and/or blindness. During acute infection parasites replicate as rapidly growing tachyzoites disseminating systemically [1]. As dissemination occurs tachyzoites differentiate into bradyzoites, forming tissue cysts in brains and muscles [2]. Differentiation is probably driven by stress conditions such as the host immune response to the parasite [3,4,5]. Current treatments cannot eliminate persistent cysts, and effective vaccines are not available [6]. As an obligate intracellular parasite, *T. gondii* utilizes readily available nutrients in its host cell and is sheltered by the host cell plasma membrane from the host’s neutralizing antibodies. Mammals are, however, armed with various innate immune mechanisms to recognize intracellular pathogens that have been shaped through millions of years of survival refined by natural selection. Parasites, on the other hand, are equipped with a plethora of immunomodulatory effectors that prevent the innate immune reaction and allow them to establish infection and shift polarization from effective responses to those that favor the parasite. In this mini review, we will go over the major *T. gondii* immunomodulators and their role(s) in infection.

*T. gondii* enters host cells by an active invasion mechanism involving the formation of a moving junction and actin-based motility [7,8]. There are three specialized vesicular organelles serially secreted by the parasite, namely micronemes, rhoptries, and dense granules [9]. In the initial attachment phase of the parasite, the micronemes deliver the adhesins such as apical membrane antigen 1 (AMA1) and microneme protein 2 to the parasite plasma membrane. Subsequently, the parasite injects rhoptry-derived vesicles into the host cytosol, which forms a complex on the host membrane, the anchoring complex [10,11], that enables the parasite to create a tight connection, the moving junction, between two membranes and propel itself into the host cell. This invasion process is rapid, with completion within 15 to 30 s [12]. Rhoptry secretion delivers not only those structural proteins that form the moving junction but also injects a variety of effectors that alter the intracellular host cell environment, making it more conducive to parasite growth. These rhoptry proteins (ROPs) include active kinases and pseudokinases that localize to the parasitophorous vacuole membrane (PVM), leading to the neutralization of host cell innate effectors, and to the nucleus leading to alterations in host cell gene expression. After *T. gondii* invades its host cell, it dissociates from the plasma membrane and forms a parasitophorous vacuole in which it will replicate. Into its parasitophorous vacuole, *T. gondii* secretes another class of secretory vesicles, dense granules, to modify the vacuolar matrix and PVM. The parasitophorous vacuole contains various dense granule proteins (GRAs) that form structures such as an intravacuolar network (IVN) for nutrient acquisition, translocons for molecular transport into the host cell, and upon bradyzoite differentiation, a cyst wall that protects parasites during latent (chronic) infection. In addition to their structural role, specific GRAs can also localize to the PVM and host nucleus modulating host cell immune reactions.

Rodents, often preyed on by felines, are the natural intermediate hosts of *T. gondii* and they have evolved various parasite-specialized innate immune mechanisms through intimate co-evolution to control *T. gondii* infection. In contrast, humans are accidental intermediate hosts in which parasites do not transmit to the next host. Anti-*T. gondii* cellular responses may display differences between the natural intermediate hosts (rodents) and accidental hosts (humans and other mammals) [13]. *T. gondii* infection in mice and humans is primarily controlled by the IFNγ pathway. Mice are a commonly utilized experimental model for studies on the immunobiology of *T. gondii* infection. Following phagocytosis of parasites, profilin, an essential cytoplasmic actin-binding protein, which aids in *T. gondii’s* gliding motility and invasion, is detected by the endo-lysosomal compartment-residing pattern recognition receptors TLR11/12 [14,15] in dendritic cells (DCs). The DCs, in turn, secrete IL-12 and activate NK cells and T cells to secrete IFNγ. IFNγ induces a cell-autonomous immune reaction that recruits immunity-related GTPases (IRGs) to the PVM [16,17,18], subsequently perforates the PVM, and destroys the parasites in the vacuole. Due to the dominance of this immune reaction in mice, *T. gondii* has evolved a plethora of immunomodulators that interfere with the IFNγ/IRG pathway. While mice have expanded the repertoire of IRGs into 23 genes in their genome, humans have only one full-length IRG gene containing a testis-specific promoter, which is not interferon-inducible [17]. Unlike mice, humans have three stop codons in TLR11 and lack TLR12 [13] and thus are unable to detect *T. gondii*’s profilin. To control *T. gondii*, humans rely on various other innate mechanisms such as TLR7/8/9 [19], inflammasome sensors [20,21], and effectors such as NO [22], tryptophan starvation [23], and autophagy-related endo-lysosomal fusion [24]. Despite the lack of dominant parasite-specific receptors and effectors, IFNγ is still a critical cytokine to control human infections with *T. gondii.* IFNγ secretion by NK and T cells depends on the stimulation of IL-12, which is produced mainly by the parasite-induced NF-κB pathway.

*T. gondii* populations in Europe and North America are composed of three classical highly clonal lineages (Type I, II, and III [25]). In comparison, South American parasites are more divergent, indicating more frequent recombination events between the strains. The three classical clonal lineages have distinct differences in virulence during acute infections in laboratory mice: Type I is highly virulent, Type II is intermediate, and Type III is avirulent. Strain-specific polymorphisms found in only a few effectors appear to account for the majority of these changes in virulence seen during acute infection.

**Figure 1:**
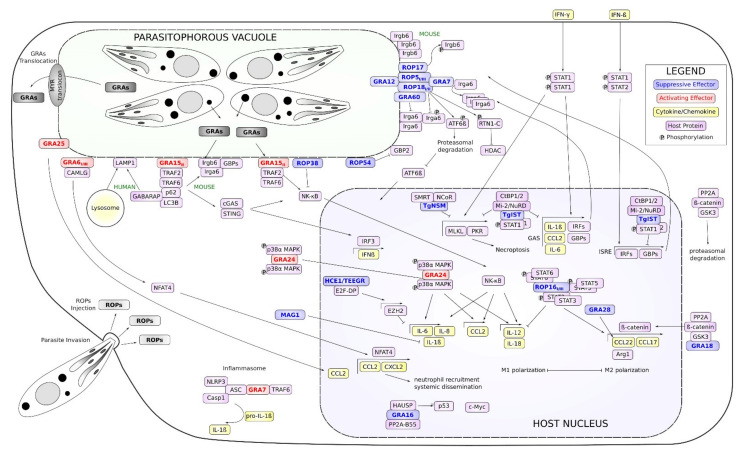
Overview of *Toxoplasma gondii* secreted immunomodulatory effectors. The schematic represents the parasite’s secreted effectors and their functions in immunomodulation. Pointed arrows indicate translocation or activation. Blunt end arrows indicate inhibition. Parasite effectors are in bold letters.

*T. gondii* has evolved various mechanisms not only to suppress, but also to promote immune reactions, in order to “fine-tune” virulence to optimize its persistence and transmission. The *T. gondii* effectors described in this review and their effects are depicted in Figure 1. In addition, the properties of these effectors and the presence of *T. gondii* orthologs in the Cystoisospora, Sarcocystis, and Eimeria genomes are listed in Table 1.

## 2. Defending Home Base: Effectors on the Parasitophorous Vacuole Membrane

### 2.1. ROP18 Virulence Complex: An Inhibitor of IFNγ-Induced IRG Loading (ROP18/ROP17/ROP5/GRA7)

IRG (or p47 GTPase) and p65 guanylate binding proteins (GBP) are IFNγ-inducible immune effectors for the acute cell-autonomous response in *T. gondii* infection [16]. In murine macrophages, IFNγ activation induces IRGs to localize on the PVM sequentially [57], hierarchical in the sequence of Irgb6, Irgb10, Irga6, Irgm2, Irgm3, and Irgd [58], and this complex perforates the PVM causing vesiculation and subsequently eliminates parasites in the vacuole [16]. To counteract IRGs, parasites secrete ROP effectors into the host cytoplasm during the invasion process, and the effectors move to the cytoplasmic surface of the PVM [29]. On the PVM the active serine-threonine kinase ROP18 [30] forms a stable complex with a pseudokinase ROP5 [29]. ROP18 phosphorylates monomeric IRGs (preferentially Irga6) on conformationally active sites, preventing their oligomerization on the PVM, and protecting parasites from IFNγ-induced killing in murine macrophages [30]. Polymorphisms of both ROP18_I/II_ [47,59] and ROP5_I/III_ [27,28] confer strain-specific acute virulence among the three classical *T. gondii* clonal lineages (Type I, II, and III), demonstrating the magnitude of the role played by the IFNγ-ROP18 virulence complex. Analogous to ROP18, an active kinase ROP17 phosphorylates polymeric Irgb6 and disassembles it into monomers and dimers [29]. The non-catalytic pseudokinase ROP5 functions as a co-factor for the ROP18 by binding to Irga6 conformationally preparing Irga6 for phosphorylation by ROP18 [60]. Deleting ROP5 renders parasites highly avirulent, while the deletion of either ROP18 or ROP17 results in a partial attenuation in acute virulence. Full avirulence was achieved with ROP17/ROP18 double mutant parasites, indicating that ROP17 and ROP18 function synergistically [29]. Compared with the ineffective variants from type II/III strain parasites, the ROP5 and ROP18 variants from the virulent type I strain can prevent IRG loading in laboratory mice. A wild-derived mouse strain CIM can prevent IRG loading in the presence of virulent ROP5_I_/ROP18_I_ variants. A polymorphic tandem IRG Irgb2-b1_CIM_ from this wild-derived CIM strain sequesters ROP5 by binding, allowing IRGs to load onto the PVM and subsequently control parasite growth [61]. A South American *T. gondii* strain VAND has a polymorphic ROP5 that escapes this Irgb2-1b_CIM_ binding enabling the parasite to prevent IRG loading and survive IFNγ-induced killing [61]. This demonstrates co-evolution between the host and pathogen, driving polymorphisms in this critical IFNγ-IRG effect. In addition to the rhoptry kinases, the dense granule proteins GRA7 [31], GRA60 [32], and potentially GRA12 [34] are also components of this ROP18 virulence complex. The dense granule protein GRA7 directly binds to ROP5 [62] to form a complex with ROP18 [31]. Although it is a dense granule protein, which is secreted much later than ROP proteins, GRA7 is recruited on the cytoplasmic surface of the PVM as early as 30 min after infection [31]. GRA7 binds to GTP-activated Irga6, inducing rapid polymerization and depolymerization [31], and subsequently reduces Irga6 loading onto the PVM [62].

### 2.2. GRA60, GRA12, ROP54: IRG/GBP Modulators

Besides the core components, the ROP18 virulence complex requires or is regulated by various additional elements. A PVM localizing dense granule protein GRA60, which appears as early as five minutes after infection, binds to ROP18 and prevents the loading of Irgb10 and Irga6, but not Irgb6 [32]. The exact mechanism of how GRA60 alters IRG loading on the PVM remains to be elucidated. GRA12, an IVN membrane-residing dense granular protein [63], is essential in preventing the IFNγ-mediated parasite killing in activated murine macrophages [34,64]. GRA12 protects parasites without stopping IRG loading on the PVM, but the effect is still dependent on Irgm, the regulatory IRGs [34]. It is speculated that the transfer of effectors from the IVN to the PVM might depend on GRA12.

ROP54 is an effector that reduces another class of GTPase effector termed GBP [33]. ROP54 reduces the IFNγ-induced GBP2 loading onto the PVM by an unknown mechanism of action. The effect is not dependent on the ROP18 virulence complex, and ROP54 does not reduce Irgb6 loading, indicating that the ROP54-GBP mechanism is specific and independent of IRGs [33]. 

### 2.3. GRA15: An Activator of NF-κB Signaling

GRA15 is a polymorphic dense granular protein that is localized to the PVM. GRA15 from type II (e.g., ME49, PLK) and III strains (e.g., CTG, VEG) induces NF-κB activation in human and mouse fibroblasts and macrophages [35,37]. Type I strains (e.g., RH strain) have a non-functional truncated GRA15 due to a premature stop codon provided by a frameshift mutation [35]. GRA15 binds to tumor necrosis factor receptor (TNFR)-associated factors 2 and 6 (TRAF2 and TRAF6) via TRAF2 binding motifs on GRA15. GRA15 induces NF-κB activation depending partially on TRAF6 [35] and TRAF2 [37], resulting in IL-12 secretion in mouse and human cells. TRAF2 is a well-characterized signaling mediator of TNF-α signal transduction through NF-κB/MAPK pathways (reviewed in [65]), resulting in various responses such as inflammation, apoptosis, and necroptosis. TRAF6 transduces IL-1R/TLR signals through NF-κB/MAPK pathways using different partners [65]. Although the exact mechanism is unknown, GRA15 usurps the signaling molecule, negating the upstream signaling input, to persistently activate NF-κB resulting in the secretion of IL-12 in human and mouse macrophages [35] and IL-1ß in human monocytes [21,66]. Both IL-12 and IL-1ß shape the early immune response towards classically activating macrophage polarization (M1). CD40, a surface receptor required for efficient macrophage activation and subsequent IL-12 secretion, is strongly upregulated by GRA15 in a NF-κB dependent manner [67]. Secreted IL-1ß also indirectly plays a critical role in protecting *T. gondii* in co-cultured hepatocytes by preventing IFNγ-induced IDO1 activation and subsequent degradation of tryptophan [68].

### 2.4. GRA15: A Mediator of IFNγ-Induced Cell-Autonomous Immune Reaction

GRA15 functions not just as an activator of NF-κB signaling, but also as a mediator of IFNγ-induced cell-autonomous immune responses in both humans and mice. The mechanism of this IFNγ-induced cell-effect is distinct in humans and mice. In human cells, such as human vasculature endothelial cells (HUVEC) and human fibroblasts (HFF), infection of IFNγ-primed cells results in the endo-lysosomal fusion of the parasitophorous vacuole and clearance [38,69], while in mouse embryonic fibroblasts (MEFs), IFNγ stimulation results in the perforation of the PVM by the immune effectors IRGs and GBPs [30]. In turn, IRGs and GBPs effectors are disarmed by the parasite’s ROP18 virulence complex in type I due to the polymorphic ROP5 and ROP18. In contrast, type II strain can actively recruit IRG/GBP to the PVM via polymorphic GRA15_II_ [46]. In MEFs, GRA15 mediates recruitment of E3 ubiquitin ligase TRAF6 to the PVM, autophagy adapter p62, and LC3B results in recruitment of Irgb6/GBPs and subsequent perforation of PVM [38]. In human cells, such as HFF, the recruitment of those autophagic adapters results in the accumulation of LAMP1 on the PVM and subsequent endo-lysosomal fusion of the parasitophorous vacuole. Surprisingly the effect of GRA15 is independent of NF-κB but dependent on the interaction of TRAF2/6 with GRA15 [38]. In addition to its role in mediation of this IFNγ effect, GRA15 also participates in the induction of IFN-ß production through cytosolic dsDNA PRR cGAS-STING in mouse cells in a partially NF-κB-independent manner [36]. These NF-κB-independent effects indicate GRA15 has multi-modal mechanisms of limiting parasite spread in the host organism.

### 2.5. ROP38: An Inhibitor of NF-κB Signaling

ROP38 is located on the PVM as well as the IVN [48,49]. It is highly expressed in type III (VEG) *T. gondii* strains but not type II (Pru) or type I (RH) strains [39,42]. A transcriptomic study revealed that ROP38 alters host cell expression, especially downregulating parasite-induced pro-inflammatory cytokines/chemokines and transcription factors such as IL-18, CXCL1, EGR2, and c-fos [42]. It has an immunosuppressive effect through inhibition of the NF-κB signaling pathway, which directly counteracts the pro-inflammatory effect of GRA15_II_ [41]. However, in the mouse infection model, deletion of ROP38 decreases IL-18 and no difference was detected in the level of IL-1ß in peritoneal fluid [39]. In one study virulence in a type II strain was reduced at a low inoculation dosage [39] and in another study was not changed at a higher inoculation dosage [40], indicating that the contribution of ROP38 to *T. gondii* virulence in mice is moderate.

### 2.6. GRA6: An Activator of NFAT4 Signaling and CCL2 Secretion

GRA6 is a membrane-associated dense granule protein located on the IVN and PVM. Together with GRA2, GRA6 stabilizes the membrane structure inside the vacuole [70]. GRA6 is also identified as a structural component of the cyst wall [71]. Besides this structural role, polymorphic GRA6 from type I, but not type II, *T. gondii* strains activates a transcription factor, the nuclear factor of activated T cells 4 (NFAT4), through its direct binding to a calcium modulating ligand (CAMLG) [43]. Activated NFAT4 translocates into the host nucleus and induces the pro-inflammatory chemokines CXCL2 and CCL2 [43]. CCL2 is a protective chemokine necessary for the recruitment of monocytes during acute *T. gondii* infection [72,73]. Contrary to the pro-inflammatory nature of this manipulation, this effect of GRA6 contributes to parasite virulence. The chemokines help recruit the CD11b+ Ly6G+ monocytes/neutrophils to the site of infection, which facilitates systemic dissemination of *T. gondii* [43].

### 2.7. GRA25: An Additional CCL2 Inducer

Similar to GRA6, a parasitophorous vacuole-residing dense granule protein GRA25 is involved in the secretion of CCL2. It was initially identified through a QTL analysis of type II/III cross progenitors that examined chemokine and cytokine secretion [44]. Deletion of GRA25 has been demonstrated to reduce pro-inflammatory cytokines such as CCL2 and CXCL1; however, GRA25 deletion also rendered the parasite avirulent. The mechanism underlying this observation might be similar to GRA6′s effect on the recruitment of immune cells for systemic dissemination.

## 3. Taking It on the Road: Effectors in the Endoplasmic Reticulum

### ROP18 and the ER

In addition to counteracting IFNγ-induced IRG/GBP loading, ROP18, the catalytic core of the ROP18 virulence complex, regulates gene expression and apoptosis. The PVM has intimate contact with host ER [74] and could affect ER functions to modulate immune reactions and stress. ROP18_I_ directly phosphorylates and subsequently degrades activating transcription factor 6ß (ATF6ß) [75]. The normal function of ATF6ß is to reside on ER membrane and upon ER stress, it is proteolytically released and translocates into the nucleus, transcribing genes involved in the unfolded protein response. Although the mechanism requires further study, suppression of unfolded protein response by ROP18 has been shown to impair antigen processing in DCs in their role in CD8 T cell stimulation [75].

Additionally, ROP18 can phosphorylate RTN1-C, an ER protein expressed in the central nervous system (CNS), leading to the induction of ER stress-mediated apoptosis in neural cells [76]. Phosphorylated RTN1-C downregulates HDAC3 (found both in the nucleus and ER) and, thereby, modulates an unfolded protein response [77]. Reduction in HDAC activity induces GRP78 acetylation, an ER-resident chaperone protein and a key regulator of the ER stress transducer. In a non-stressed cell, GRP78 binds to the PERK, IRE1, and ATF6 (activators of the unfolded protein response) and keeps them inactive [78]. GRP78 acetylation induces dissociation of PERK and resulted in ER stress-mediated apoptosis in a neuronal cell line [76]. The effect of ROP18-induced apoptosis is moderate. On that note, there is also an increase in baseline apoptosis induced by ∆*rop18* parasites, indicating that there are probably additional mechanisms independent of ROP18.

## 4. Taking It on the Road: Effectors in the Cytoplasm

### 4.1. GRA18: ß-Catenin Induced M2 Cytokines Secretion

GRA18 is translocated across the PVM into the host cytoplasm via the MYR translocon (a protein complex that mediates the transport of effector proteins which is described in detail in the section entitled “Translocation of GRA Effectors”) [45]. In the cytoplasm, GRA18 stably binds to a ß-catenin destruction complex including ß-catenin, glycogen synthase kinase-3 (GSK3), and protein phosphatase 2A regulatory subunit B56 (PP2A-B56) [45]. ß-catenin is a central signal transducer in the canonical Wnt signaling pathway. In the absence of Wnt ligands, ß-catenin is phosphorylated by the destruction complex resulting in continuous proteasomal degradation. Once the Wnt ligands engage with receptors, ß-catenin escapes from the destruction complex, translocates into the nucleus, works as a transcriptional co-activator for the Wnt-specific transcription factor, and transcribes the genes necessary for embryonic development and tissue homeostasis [79]. The parasite effector GRA18 hijacks this Wnt-ß-catenin mechanism. Typical Wnt-target genes were not induced in bone marrow-derived macrophages (BMDM); instead, a set of chemokines (transcripts and subsequent secretion), including CCL17, CCL22, and CCL24, are upregulated in a GRA18-dependent manner [45]. Both CCL17 and CCL22 bind to the CCR4 chemokine receptors and attract Th2 in inflammatory conditions [80] and T-reg cells in non-inflammatory conditions [81]. Deletion of ß-catenin in the mouse macrophage cell line RAW264.7 demonstrated a dependency on CCL17 but not on the CCL22 secretion, indicating there could be a ß-catenin-independent GRA18 effect potentially based on the GSK3 inhibition. Even though the mechanism is still unclear, deletion of GRA18 rendered parasites significantly less virulent, potentially due to the inability of the parasite to shift the immune response toward Th2/M2, indicating the importance of GRA18 in modulating this immune reaction during acute infection [45]. 

### 4.2. MAG1: An Effector, Matrix, and Cyst Wall Protein

MAG1 is a dense granule protein that localizes to the parasitophorous vacuole and PVM [82]. It has been recently shown that MAG1 is secreted beyond the PVM and into the host cytosol [46]. It is produced in the tachyzoite stage as a matrix protein, but MAG1 migrates to the cyst wall upon bradyzoite differentiation. In mouse macrophages, parasite infection induces secretion of IL-1ß in low amounts. GRA15 is a known inducer of NF-κB signaling and subsequent IL-1ß secretion in an inflammasome component-dependent manner in human monocytes (NLRP3, ASC, and caspase 1) [20,21] and in mouse macrophages (NLRP1/3, ASC, and caspase 1/11) [83,84]. However, infection with parasites that had a deletion of MAG1 significantly increased the secretion of IL-1ß from the baseline, indicating that MAG1 dampens IL-1ß secretion in mouse macrophages [46]. Inhibition of IL-1ß by MAG1 does not involve suppression of NF-κB activation, IL-12 secretion, or TNF-α secretion. Instead, GRA15 is necessary for IL-1ß secretion in ∆*mag1* parasites [46]. In the classical two-signal model of the inflammasome [85], signal 1 (priming) activates transcription factor NF-κB inducing expression of pro-IL-1ß, then signal 2 (activation) activates oligomerization of inflammasome components resulting in the processing of pro-IL-1ß into a mature form ready for secretion. While GRA15 activates signal 1 through NF-κB, MAG1 might be inhibiting some circuits in signal 2, resulting in the selective inhibition of GRA15 downstream effects. Unlike other dense granule proteins, MAG1 cytoplasmic translocation does not require the MYR1 translocon. The majority of MAG1 is retained in the parasitophorous vacuole and PVM. Whether MAG1 needs to be translocated into the host cytoplasm to inhibit IL-1ß or its localization on the PVM is sufficient for this effect has not been determined. 

## 5. Attacking Enemy Headquarters: Effectors Found in the Host Nucleus

### 5.1. ROP16: An Inducer of M2 Polarization

ROP16 is a polymorphic kinase secreted from the rhoptry organelle which has been demonstrated to determine virulence in a *T. gondii* strain-specific manner [47,48]. ROP16 is injected into the host cytoplasm during the invasion process, and it translocates into the host cell nucleus within 10 min [86]. ROP16_I/III_ directly phosphorylates transcription factors STAT3 and STAT6 [87,88], which induce T-helper 2 (Th2) polarization [89]. The activation kinetics of STAT6 was much quicker with ROP16 (1 min from invasion) than with IL-4 treatment (5 min from stimulation) which is a natural inducer of STAT6. While the negative feedback by STAT6 target transcripts usually deactivates STAT6 through deactivation of the upstream JAK kinase, the ROP16-induced STAT6 pathway bypasses these upstream activators, resulting in persistent activation of STAT6 [87]. Most ROP16 in the host cell is found in the nucleus due to its nuclear localization signal (NLS); however, this NLS is dispensable for ROP16 activation of STAT3/6 [86], indicating that ROP16 probably phosphorylates STAT3/6 in the cytoplasm and is subsequently translocated into the nucleus. STAT6 phosphorylation by ROP16_I/III_ shifts the activation of macrophages toward an alternatively activated state (M2) instead of the classically activated state (M1), as is evident by the various M2 signatures that are seen such as arginase-I activity and suppression of IL-12 secretion [90]. The anti-inflammatory effect of M2 polarization by ROP16_I/III_ reduces the mortality of an otherwise lethal intestinal inflammatory response in vivo models [48]. This protective effect has been shown to be transduced through STAT5 instead of STAT6 and required GRA15′s effect as a Th1 immune activator.

### 5.2. GRA28: An Inducer of CCL22 Chemokine Secretion

GRA28 is a dense granule protein localized to the host nucleus in a MYR-dependent manner [91]. A recent pre-print described that GRA28 could induce the secretion of the M2 macrophage-associated chemokine CCL22 [49], which is known to recruit regulatory T cells in tumor microenvironments [80,81]. Unlike GRA18, which induces secretion of both CCL22 and CCL17, GRA28 only induces CCL22 without affecting CCL17 or any other M2-associated signaling molecules. Although there were no differences in mortality or morbidity, *∆gra28* parasites had a significant reduction in brain cyst burden. It is possible that the immunosuppressive effect of GRA28 might be necessary for brain cyst persistence.

### 5.3. GRA24: A Modulator of p38 α Mitogen-Activated Protein

GRA24 is an intrinsically disordered dense granule protein translocated to the host nucleus [50] through the MYR translocon [92]. Compared with the ROP proteins injected during initial invasion, GRA24 appears in the host nucleus starting at 16 h post-invasion. GRA24 forms a stable complex with two independent copies of host p38α mitogen-activated protein (MAP) kinases via two kinase interaction motifs on GRA24, sequestering p38α from its endogenous regulatory proteins [93]. Compared with natural activation that happens transiently, this GRA24-induced p38α activation is sustained for an extended period. This sustained activation could be due to the high-affinity binding of GRA24 to p38α, which in turn prevents deactivation by endogenous regulatory phosphatases. The hyperactive GRA24-p38α complex activates natural targets such as activating transcription factor 2 (ATF2) [93]. Subsequently, it activates other transcription factors such as c-fos and EGR1 (the early response genes) [93], resulting in the transcription of pro-inflammatory cytokine and chemokine genes (e.g., IL-6, IL-12p40, TNF, CCL2, CCL5, CXCL1, and CXCL10) [50]. More prominently in type II parasites than in type I parasites, M1 response-associated genes are upregulated, while M2 genes are downregulated in a GRA24-dependent manner [50]. Although both GRA15 and GRA24 induce the secretion of IL-12, their activation mechanisms do not overlap with each other. GRA15 exclusively activates NF-κB, while GRA24 activates p38α exclusively [50], ensuring immune activation and fine-tuning of this response. A recent MyD88-deficient mouse in vivo study demonstrated that through p38 activation, GRA24 induced IL-12 production in macrophages, neutrophils, and dendritic cells independent of the profilin/TLR11/MyD88 signaling pathway, resulting in the IFNγ production and a Th1 response [94] and enabling GRA24 to protect the host from an otherwise lethal challenge [94]. Similarly, an in vivo study using a TLR11-deficient mouse demonstrated that both GRA24 and GRA15 shape the early immune response towards Th1/M1 [95].

### 5.4. GRA16: A Modulator of HAUSP and PP2A

GRA16 translocates into the host nucleus, is detectable four hours post-infection, and peaks at 24 h post-infection. It forms a stable complex with the ubiquitin-specific protease HAUSP and the B55 regulatory subunit of protein phosphatase 2A (PP2A-B55) [51]. HAUSP is a known regulator of a tumor suppressor p53 protein stability. In normal cells, p53 is maintained at a low level by ubiquitination and proteasomally degraded. HAUSP cleaves ubiquitins and subsequently stabilizes p53 [96]. Thus, *T. gondii* infection stabilizes p53 in a GRA16 dependent manner. GRA16 binding of PP2A-B55 also induces a cytoplasm to nuclear translocation of the PP2A holoenzyme. Ectopic expression of GRA16 in tumor cells demonstrated the GRA16 could induce a p53-dependent pro-apoptotic effect [97] and PP2A-dependent suppression of NF-κB signaling via inhibition of upstream AKT signaling [98]. However, it remains to be examined if these immunomodulatory effects which were observed in parasite-free tumor cell models are relevant mechanisms during infection. The GRA16-activated PP2A holoenzyme also upregulates c-Myc expression and nuclear accumulation [99] collaboratively with other unknown MYR-dependent effectors (potentially GRA24 and HCE1/TEEGR). 

### 5.5. HCE1/TEEGR: A Modulation of E2F Activation

HCE1 (host cyclin E 1)/TEEGR (E2F4-associated EZH2-inducing gene regulator) is an intrinsically disordered dense granule protein that translocates across the PVM via MYR1 dependent mechanism into host cell nuclei [52,53]. HCE1/TEEGR forms a complex with transcription factors E2F3/4/DP1/2 [52]. E2Fs are major transcriptional regulators of cell cycle-dependent gene expression [100]. This HCE1/TEEGR-dependent E2F activation induces the expression of Cyclin E, which is required for the G1/S cell cycle transition [53]. This E2F activation also induces the expression of EZH2, which is the catalytic subunit of polycomb repressive complex 2 (PRC2) that drives epigenetic silencing by histone methyltransferase activity (H3K27me3) [52]. Certain host transcription factors can recruit PRC2 to the CpG islands and add the repressive histone markers [101]. HCE1/TEEGR usurps this strategy by promoting recruitment of EZH2 (and probably the PRC2) to the NF-κB-regulated genes loci, including IL-6 and IL-8 loci resulting in the H3K27me3 modification. This epigenetic silencing subsequently reduces the transcription of IL-6, IL-8, and IL-1ß and dampens NF-κB activation. Suppression of NF-κB activation by HCE1/TEEGR is not thorough since the WT parasite can still induce NF-κB translocation comparable to TNFα treatment. Deletion of HCE1/TEEGR significantly reduces virulence, demonstrating its importance in acute infection.

### 5.6. TgIST: A Suppressor of Interferon Signaling

TgIST (*T. gondii* inhibitor of STAT transcription) is a highly disordered dense granule protein translocating into the host nucleus (it is visible at 9 h after tachyzoite invasion but diminished at 48 h) [54]. TgIST suppresses both Type II IFN (IFNγ) [54] and Type I IFN (IFNα/ß) signal transduction [102]. The IFNγ response is essential for controlling parasites in mice [103], while IFNα/ß has modest importance in the protective role [104]. In the normal response to IFNγ stimulation, STAT1 (a transcription factor) forms homodimers, binds to the GAS-(IFNγ activated site) sequence containing promoters, and induces the transcription of pro-inflammatory genes, including IFNγ-inducible IRGs. TgIST hijacks this IFNγ signaling pathway by forming a stable complex with activated STAT1, the chromatin repressor Mi-2-nucleosome remodeling deacetylase (Mi-2/NuRD) complex, and the co-repressors C-terminal-binding proteins (CtBP1/2) [54,55]. In normal cellular functions without *T. gondii* infection, STAT1 is not associated with the NuRD complex [55], which possesses both ATP-dependent nucleosome remodeling and histone deacetylase capabilities essential for gene silencing in development and cellular differentiation [105]. This repressive TgIST/STAT1 complex persists on the STAT1 binding sites with GAS sequences remodeling the chromatin environment (by H3K4me3 marks on promoter) suppressing the transcription of various pro-inflammatory IFNγ-induced cytokines and chemokines. TgIST prevents IFNγ-stimulated IRG loading onto the PVM, protecting parasites from the cell-autonomous immune response and subsequent destruction. In vivo, deletion of TgIST protected mice from an otherwise lethal dose of parasite challenge in a type I IFN receptor-dependent manner [54,55]. This difference was attributed to early Gr1+ inflammatory monocyte recruitment to the peritoneal cavity. In distinction from IFNγ signal transduction, the type I IFNs induce STAT1 and STAT2 heterodimer formation and the activated STAT1/2 dimer binds to the interferon-sensitive response element (ISRE) sequence in promoters and drives transcription of pro-inflammatory genes. TgIST also suppresses type I (IFN-ß) signal transduction by binding to the STAT1/2 heterodimer with the aforementioned NuRD complex [102]. Unlike the parasiticidal IFNγ stimulation, IFN-ß stimulation results in relatively milder growth restriction in mouse and human cells. TgIST prevents this IFN-ß-induced growth inhibition which is dependent on both STAT1 and type I IFN receptor [102]. The effect of TgIST on type I IFN in an in vivo murine model revealed that the reduced virulence exhibited by the ∆*Tgist* parasites was restored by deletion of the type I IFN receptor in the mouse, indicating that suppression of type I IFN signaling plays a role in mouse pathology.

### 5.7. TgNSM: A Suppressor of IFNγ-Induced Necroptosis

TgNSM (NCoR/SMAT modulator) is a dense granule protein that is translocated into the host cell nucleus and suppresses the IFN signals [56]. Unlike other nuclear effectors that significantly diminish nuclear translocation in bradyzoites [106,107], TgNSM is detectable in the bradyzoite stage even at five days after infection [56]. Once in the host nucleus, TgNSM binds to the nuclear receptor co-repressor/silencing mediator for retinoid and thyroid hormone receptors (NCoR/SMRT) transcriptional co-repressor complex [56]. In normal cellular function, this NCoR/SMRT complex binds to various un-liganded nuclear receptors such as estrogen and androgen receptors and represses unintended non-specific transcriptions of the target genes [108]. However, this parasite-hijacked TgNSM-NCoR/SMRT complex can inhibit specific IFNγ induced genes, including crucial genes (e.g., RIPK1, RIPK3, MLKL, and PKR) regulating the IFNγ induced necroptotic death pathway. Necroptosis is a form of regulated cell death mechanism initiated by binding of IFNγ, type I IFNs, TNF, and LPS to receptors, which induces a necrosome complex formation and subsequent MLKL oligomerization, resulting in plasma membrane permeabilization [109]. In the bradyzoite stage, *T. gondii* can prevent this IFN-induced necroptosis by transcriptional repression of PKR and MLKL via the combinatorial effect of both TgNSM and TgIST. Neither ∆*Tgnsm* nor ∆*Tgist* single-deletion mutants were susceptible to IFN-induced necroptosis, indicating the redundancy in repressed targets. 

## 6. Translocation of GRA Effectors

There is a recent systematic review on effector translocation [110]; therefore, we will focus on updating the information in that review. A recently identified molecular chaperone GRA45, which has structural homology to the α-Crystallin domain of small heat shock protein, is necessary for transporting effector GRAs through the secretory pathway [111]. When GRA45 is deleted in *T. gondii,* other dense granule proteins (e.g., GRA5, GRA7, MAF1, GRA23, GRA16, and GRA24) are mislocalized to the vacuolar matrix and fail to be transported to the PVM and beyond [111]. As a result of this dense granule effector transport failure, ∆*gra45* parasites are incapable of preventing IFNγ-mediated growth inhibition in vitro and demonstrate reduced virulence in vivo. 

Many dense granule effectors require translocation across the PVM when secreted into the host cell cytosol (GRA18) or nucleus (GRA16, GRA24, GRA28, HCE1/TEEGR, TgIST, and TgNSM). On the PVM, the parasite forms a GRA17/GRA23 small-molecule translocon that mediates the passive transport of small molecules [112]. This GRA17/23 translocon can pass 500 Da but not 3000 Da molecules, indicating that this complex cannot transport the large dense granule effectors. To translocate these dense granule proteins (GRAs) *T. gondii* has evolved a specialized translocon machinery. This translocon was identified by screening for parasite effectors that could upregulate host c-Myc; the translocon identified has been named the MYR (Myc regulation) translocon and is composed of three transmembrane-containing dense granule proteins: MYR1 [92], MYR2, and MYR3 [113]. As with many other dense granule proteins, MYR1 is proteolytically cleaved into two fragments by a Golgi-associated aspartyl protease 5 (ASP5) on a TEXEL motif (Toxoplasma Export Element, RRLxx). ASP5-processing of MYR1 and many other dense granule effectors is necessary for their proper function. The MYR translocon is required for the translocation of several GRA nuclear-targeted protein effectors (e.g., GRA16 and GRA24) and cytoplasmic protein effectors (e.g., GRA18), but not for PVM localized effectors (e.g., GRA15) [92]. The exact mechanism of GRA translocation through the MYR translocon is not known; however, generally speaking, translocated GRA effectors are intrinsically disordered. The addition of a highly structured domain DHFR to GRA16 prevented its translocation [113], indicating that an unfolding process is involved in the translocation mechanism. 

In addition to the MYR translocon, ROP17, the secreted kinase that phosphorylates IRGs, is also necessary for the translocation of GRAs [114], probably via the phosphorylation of translocon-associated proteins. Other post-translational modifications are also necessary for the export of GRA effectors. TgPPM3C is a dense-granule phosphatase located on the cyst wall [115]. Phospho-proteomic evidence demonstrates that TgPPM3C likely dephosphorylates effectors such as GRA16 and GRA28 [116] and this affects their translocation across the PVM. Deletion of TgPPM3C reduces the translocation of GRA16 and GRA28, but not the GRA24 or TgIST, rendering the parasite highly avirulent. Although they are not effectors by themselves, the chaperone, Golgi-resident protease, the translocons, and post-translational regulators are required for GRA effector functions by ensuring the transport of the effectors to the proper sites of targets. 

## 7. Effector Translocation in *T. gondii* Tissue Cysts

When *T. gondii* senses immunological stress such as IFNγ [48] and NO [4], they differentiate from fast-growing tachyzoites to slow-growing bradyzoites. Bradyzoites form tissue cysts in the host and persist for an extended period, providing an opportunity for transmission to the next host as well as development via sexual reproduction if tissue cysts are ingested by felines. During chronic infection the parasite must control the immune response to survive. Upon bradyzoite differentiation, parasites within the parasitophorous vacuole generate a cyst wall composed of the PVM and an amorphous granular layer with abundant O-GalNAc glycans that is beneath the limiting PVM [117]. A proteomic study of the cyst wall identified MYR1 as a component of this structure [115]. Since dense granule translocation across the PVM is mediated by the MYR translocon [92,113], it is reasonable to speculate that immunomodulatory effectors are secreted into the host cell across the cyst wall.

Contrary to what was observed in the kinetics of GRA effector translocation in host cells containing tachyzoite parasitophorous vacuoles, the nuclear effectors GRA16, GRA24, GRA28, and TgIST had significantly diminished translocation in host cells that contained bradyzoite parasitophorous vacuoles [106,107]. Whereas GRA24 and GRA28 had no detectable translocation, TgIST was detectable four days after infection [106]. The reduction in effector export was not due to a decrease in GRA expression in bradyzoites as these effectors were still present in the vacuole [106]. Even when overexpressed with an inducible promoter, there was no significant increase in GRA16 and GRA24 translocation [107], indicating that the cyst wall has different translocation kinetics than the tachyzoite PVM and might have distinct modifications to the translocon structure at its PVM. 

Unlike other nuclear effectors, the newly discovered TgNSM is robustly translocated in both tachyzoite and bradyzoite stages. Either TgNSM or TgIST can prevent necroptosis of cysts [56], suggesting that the low level of TgIST nuclear translocation observed in cysts is still functional. It also demonstrates the functional redundancy of these effectors and the importance of suppressing IFNγ signaling during the chronic stage of infection.

## 8. Perspectives

This survey of known secreted immunomodulators demonstrates a cornucopia of intricate immunomodulation mechanisms. These modulators do not seem to have an apparent common theme in their mechanism of action, but instead, they represent diverse variations. For example, among the nuclear effector proteins: ROP16_I/III_ directly commandeers transcription factors and alters gene regulation; TgIST usurps transcription factors with additional unrelated chromatin remodeling repressors and silences the target genes; and TgNSM hijacks a chromatin repressor complex that normally maintains a specific subset of pro-inflammatory genes. All three genes are not evolutionarily related yet perform similar transcriptomic manipulations. What is noticeable is the sheer number of immunomodulatory effectors that interfere with the IFNγ-mediated GTPase responses. The nuclear effectors TgIST and TgNSM suppress IFNγ signaling at the transcriptomic level, while ROP54 and the ROP18/5/7/GRA7 virulence complex with ancillary GRA60/12 proteins block downstream GTPases loading on the PVM. We believe that the fierce selective pressure on *T. gondii*’s immunomodulatory effectors and murine immune-effectors is evident in the co-evolution of a wild-derived murine Irg2b that resists the effect of ROP18_I_/ROP5_I_ and a South American ROP5 that deactivates the resistant Irg2b [61]. The other major parasite-targeted immunological pathway is the NF-κB pathway. ROP16, HCE1/TEEGR, ROP38 all suppress at least a subset of NF-κB controlled genes, while GRA15 and GRA24 activate NF-κB targets. This demonstrates an important role of NF-κB signaling in *T. gondii* infection as well as the need for both the activators and suppressors for fine adjustment of immune subversion and parasite clearance during this infection. 

Scientific technologies have been advancing rapidly, and the available analytical methods also have shaped how we identify immunomodulatory effectors. In the early 2000s, the use of genetic crosses between clonal lineages and QTL mapping enabled the identification of various virulence determining effectors such as ROP16, ROP18 [47], ROP5 [27,28], and GRA15 [35]. Complete genome sequencing has allowed us to identify a list of potential secreted effectors based on the presence of a signal peptide, intrinsic disorder, and NLS. This in silico approach identified various nuclear effectors such as GRA16 [51], GRA24 [50], TgIST [54], and HCE1/TEEGR [52]. Recent advancements have enabled the use of CRISPR-based phenotypic screening, which resulted in the identification of GRA45 [111]. The proteomic approach using proximity labeling of nuclear protein has identified existing and novel nuclear effectors, including TgNSM [56]. Single-cell RNA sequencing has facilitated a higher resolution image of parasite–host interactions [118,119,120].

Essential questions that remain to be answered are: how these effectors exert their effects in concert; how GRA translocations across the PVM are regulated by the MYR translocon; and how effectors contribute to tissue cyst persistence. Effectors do not exert their effects in isolation; rather, multiple effectors can work synergistically, such as TgIST and TgNSM [56], or antagonistically, such as GRA15 and ROP16 [48]. In a recent study, clever use of a MYR1 deletion mutant allowed an examination of the bulk effects of all ROPs (ROP-injected but not infected population), MYR-dependent GRAs (wild-type infected), or MYR-independent GRAs (∆*myr1*-infected) [118]. More flexible and manipulatable control of effector expression could enable us to observe the combinatorial effect of multiple effectors on immunomodulation. The kinetics of various effector translocation are regulated differently among the exported GRA effectors. Phosphorylation [116], proteolytic cleavage [121,122], and intrinsic disorder [113] influence their export, but how this is spatiotemporally regulated still needs further research. Last but not least is the immunomodulation observed during the chronic stage of infection. Tissue cysts have to hide from immune surveillance while maintaining their survival in the host organism. The amount of translocation of the described effectors dwindles in the bradyzoite stage [106], with the exception of TgNSM [56]. If other bradyzoite-specific effectors exist is unknown. Whether the immunomodulation during early infection by various effectors and the persistent TgNSM is sufficient to keep cysts under the “immunologic” radar is not known. Understanding how cysts evade immune reactions would enable us to break cyst immune tolerance to target and ultimately eradicate cysts from chronically infected people.

## Figures and Tables

**Table 1:** Secreted effectors identified in *Toxoplasma gondii*.

Name	Location	Functions	Interacting Partner	Mouse Virulence of KO Parasites	Gene Name	Ortholog in Cystoisospora	Ortholog in Sarcocystis	Ortholog in Eimeria	Essentiality Score [26]	NonSyn/Snyn SNP	References
ROP5_I/III_	PVM	Pseudokinase inhibits IRG coating by preparing Irga6 for phosphorylation by ROP18.	Irga6	Highly avirulent [27,28]	TGME49_308090	Y	Y	Y	n/a	3	[27,28]
ROP18_I/II_	PVM/ER	Serine-threonine kinase inhibits IRG coating by phosphorylating monomeric Irga6. Suppresses unfolded protein response and antigen presentation. Induces apoptosis in neurons by RTN1-C.	IRGs (preferentially Irga6), ATF6ß, RTN1-C	Slightly reduced, ∆*rop18/17* double mutant is highly avirulent [29]	TGME49_205250	Y	Y	Y	0.89	4.5	[30]
GRA7	PVM	Alter the turnover kinetics of Irga6. Activate inflammasome.	Irga6, TRAF6, ASC	Slightly reduced, ∆*rop18/gra7* double mutant is highly avirulent [31]	TGME49_203310	N	N	N	1.9	4	[31]
ROP17	PVM	Serine-threonine kinase disassembles polymerized Irgb6.	IRGs (preferentially Irgb6)	Slightly reduced, ∆*rop18/17* double mutant is highly avirulent [29]	TGME49_258580	Y	Y	Y	1.29	3.8	[29]
GRA60	PVM	Prevents Irga6 and Irgb10 coating on PVM.	n/a	Slightly reduced [32]	TGME49_204270	N	N	N	-0.59	2.41	[32]
ROP54	PVM	Reduces GBP2 loading on PVM.	n/a	Reduced [33]	TGME49_210370	N	N	N	1.07	1.58	[33]
GRA12	IVN/PVM	Prevents IFNγ-induced parasite destruction.	n/a	Highly avirulent [34]	TGME49_288650	Y	Y	Y	1.75	0.64	[34]
GRA15_II_	PVM	Activates NF-κB, induce secretion of IL-12 and IL-1ß. Mediates IFNγ-induced PV-lysosome fusion and IRG loading.	TRAF2/TRAF6	No difference [35], more virulent [36]	TGME49_275470	N	N	N	2.33	3.75	[35,37,38]
ROP38	IVN/PVM	Inhibits NF-κB pathway and IL-12 production.	n/a	Reduced [39], no difference [40]	TGME49_242110	Y	Y	Y	n/a	4.5	[41,42]
GRA6	IVN/PVM	Induces CXCL2/CCL2 by activation NFAT4, recruits monocytes/neutrophils, and disseminates parasites.	CAMLG	Reduced [43]	TGME49_275440	N	N	N	1.84	8.25	[43]
GRA25	PV	Induces CCL2 secretion.	n/a	Reduced [44]	TGME49_290700	N	N	N	1.31	3.08	[44]
GRA18	Cytoplasm	Activates ß-catenin and release anti-inflammatory chemokines CCL22 and CCL17.	GSK3/PP2A-B56	Reduced [45]	TGME49_288840	N	N	N	1.66	1.93	[45]
MAG1	PVM/Cytoplasm	Suppresses IL-1ß secretion in macrophages.	n/a	Reduced [46]	TGME49_270240	Y	N	N	1.25	1	[46]
ROP16_I/III_	Nucleus	Suppresses Th1/M1 response, induces Th2/M2 response. Protects mice from lethal ileitis.	STAT3/5/6	More virulent [47,48]	TGME49_262730	N	N	N	1.11	2.37	[47,48]
GRA28	Nucleus	Induces CCL22 secretion.	n/a	No difference [49]	TGME49_231960	N	N	N	1.48	5.25	[49]
GRA24	Nucleus	Activates p38α MAPK and induces Th1/M1 cytokines and chemokines secretion (e.g., IL-12, IL-6, CCL2, CCL5).	p38α MAPK	No difference [50]	TGME49_230180	N	N	N	2.86	2.69	[50]
GRA16	Nucleus	Interferes p53-dependent apoptosis and inhibits NF-κB pathway (in parasite-free tumor model).	HAUSP, PP2A-B55	Reduced in type II strain but not in type I [51]	TGME49_208830	N	N	N	2.28	3.13	[51]
HCE1/TEEGR	Nucleus	Represses NF-κB-induced gene expression (e.g., IL-6, IL-8, IL-1ß).	E2F	Reduced [52]	TGME49_239010	N	N	N	1.46	5.46	[52,53]
TgIST	Nucleus	Represses IFN-induced gene expression (e.g., IRGs loading).	STAT1/2, NuRD	Highly avirulent [54,55]	TGME49_240060	N	N	N	1.44	5.67	[54,55]
TgNSM	Nucleus	Represses IFN-induced gene expression and necroptosis.	NCoR/SMRT	n/a	TGME49_235140	N	N	N	1.41	6.73	[56]

The table represents the immunomodulatory secreted effectors and their properties. The gene names are based on the ME49 strain. Y in the ortholog section indicates the presence of at least one ortholog in the corresponding genus, and N indicates absence. Orthologs and non-synonymous/synonymous SNP ratios are curated based on the ToxoDB release 53. n/a = data not available.

## Data Availability

Not applicable.

## Abbreviations

EZH2	enhancer of zeste homolog 2
GBP	p65 guanylate binding protein
IRG	immunity-related GTPase
IVN	intravacuolar network
NF-κB	nuclear factor kappa B
NLS	nuclear localization signal
PVM	parasitophorous vacuolar membrane
QTL	quantitative trait locus
STAT	signal transducer and activator of transcription
TRAF	TNF receptor-associated factor

## References

[1] Derouin F., Garin Y.J. (1991). *Toxoplasma gondii*: Blood and tissue kinetics during acute and chronic infections in mice. Exp. Parasitol..

[2] Di Cristina M., Marocco D., Galizi R., Proietti C., Spaccapelo R., Crisanti A. (2008). Temporal and spatial distribution of *Toxoplasma gondii* differentiation into Bradyzoites and tissue cyst formation in vivo. Infect. Immun..

[3] Bohne W., Heesemann J., Gross U. (1993). Induction of bradyzoite-specific *Toxoplasma gondii* antigens in gamma interferon-treated mouse macrophages. Infect. Immun..

[4] Bohne W., Heesemann J., Gross U. (1994). Reduced replication of *Toxoplasma gondii* is necessary for induction of bradyzoite-specific antigens: A possible role for nitric oxide in triggering stage conversion. Infect. Immun..

[5] Weiss L.M., Laplace D., Takvorian P.M., Tanowitz H.B., Cali A., Wittner M. (1995). A cell culture system for study of the development of *Toxoplasma gondii* bradyzoites. J. Eukaryot. Microbiol..

[6] Elsheikha H.M., Marra C.M., Zhu X.-Q. (2021). Epidemiology, Pathophysiology, Diagnosis, and Management of Cerebral Toxoplasmosis. Clin. Microbiol. Rev..

[7] Mordue D.G., Desai N., Dustin M., Sibley L.D. (1999). Invasion by *Toxoplasma gondii* establishes a moving junction that selectively excludes host cell plasma membrane proteins on the basis of their membrane anchoring. J. Exp. Med..

[8] Sibley L.D. (2004). Intracellular parasite invasion strategies. Science.

[9] Carruthers V.B., Sibley L.D. (1997). Sequential protein secretion from three distinct organelles of *Toxoplasma gondii* accompanies invasion of human fibroblasts. Eur. J. Cell Biol..

[10] Lebrun M., Michelin A., El Hajj H., Poncet J., Bradley P.J., Vial H., Dubremetz J.F. (2005). The rhoptry neck protein RON4 re-localizes at the moving junction during *Toxoplasma gondii* invasion. Cell. Microbiol..

[11] Alexander D.L., Mital J., Ward G.E., Bradley P., Boothroyd J.C. (2005). Identification of the moving junction complex of *Toxoplasma gondii*: A collaboration between distinct secretory organelles. PLoS Pathog..

[12] Morisaki J.H., Heuser J.E., Sibley L.D. (1995). Invasion of *Toxoplasma gondii* occurs by active penetration of the host cell. J. Cell Sci..

[13] Gazzinelli R.T., Mendonça-Neto R., Lilue J., Howard J., Sher A. (2014). Innate Resistance against *Toxoplasma gondii*: An Evolutionary Tale of Mice, Cats, and Men. Cell Host Microbe.

[14] Yarovinsky F., Zhang D., Andersen J.F., Bannenberg G.L., Serhan C.N., Hayden M.S., Hieny S., Sutterwala F.S., Flavell R.A., Ghosh S. (2005). TLR11 activation of dendritic cells by a protozoan profilin-like protein. Science.

[15] Raetz M., Kibardin A., Sturge C.R., Pifer R., Li H., Burstein E., Ozato K., Larin S., Yarovinsky F. (2013). Cooperation of TLR12 and TLR11 in the IRF8-dependent IL-12 response to *Toxoplasma gondii* profilin. J. Immunol..

[16] Taylor G.A., Feng C.G., Sher A. (2007). Control of IFN-gamma-mediated host resistance to intracellular pathogens by immunity-related GTPases (p47 GTPases). Microbes Infect..

[17] Bekpen C., Hunn J.P., Rohde C., Parvanova I., Guethlein L., Dunn D.M., Glowalla E., Leptin M., Howard J.C. (2005). The interferon-inducible p47 (IRG) GTPases in vertebrates: Loss of the cell autonomous resistance mechanism in the human lineage. Genome Biol..

[18] Butcher B.A., Greene R.I., Henry S.C., Annecharico K.L., Weinberg J.B., Denkers E.Y., Sher A., Taylor G.A. (2005). p47 GTPases regulate *Toxoplasma gondii* survival in activated macrophages. Infect. Immun..

[19] Andrade W.A., Souza M.D.C., Ramos-Martinez E., Nagpal K., Dutra M.S., Melo M.B., Bartholomeu D.C., Ghosh S., Golenbock D.T., Gazzinelli R.T. (2013). Combined Action of Nucleic Acid-Sensing Toll-like Receptors and TLR11/TLR12 Heterodimers Imparts Resistance to *Toxoplasma gondii* in Mice. Cell Host Microbe.

[20] Gov L., Schneider C.A., Lima T.S., Pandori W., Lodoen M.B. (2017). NLRP3 and Potassium Efflux Drive Rapid IL-1β Release from Primary Human Monocytes during *Toxoplasma gondii* Infection. J. Immunol..

[21] Gov L., Karimzadeh A., Ueno N., Lodoen M.B. (2013). Human innate immunity to *Toxoplasma gondii* is mediated by host caspase-1 and ASC and parasite GRA15. mBio.

[22] Nagineni C.N., Pardhasaradhi K., Martins M.C., Detrick B., Hooks J.J. (1996). Mechanisms of interferon-induced inhibition of *Toxoplasma gondii* replication in human retinal pigment epithelial cells. Infect. Immun..

[23] Pfefferkorn E.R. (1984). Interferon gamma blocks the growth of *Toxoplasma gondii* in human fibroblasts by inducing the host cells to degrade tryptophan. Proc. Natl. Acad. Sci. USA.

[24] Andrade R.M., Wessendarp M., Gubbels M.-J., Striepen B., Subauste C.S. (2006). CD40 induces macrophage anti–*Toxoplasma gondii* activity by triggering autophagy-dependent fusion of pathogen-containing vacuoles and lysosomes. J. Clin. Investig..

[25] Howe D.K., Sibley L.D. (1995). *Toxoplasma gondii* comprises three clonal lineages: Correlation of parasite genotype with human disease. J. Infect. Dis..

[26] Sidik S.M., Huet D., Ganesan S.M., Huynh M.-H., Wang T., Nasamu A.S., Thiru P., Saeij J.P.J., Carruthers V.B., Niles J.C. (2016). A Genome-wide CRISPR Screen in Toxoplasma Identifies Essential Apicomplexan Genes. Cell.

[27] Behnke M.S., Khan A., Wootton J.C., Dubey J.P., Tang K., Sibley L.D. (2011). Virulence differences in Toxoplasma mediated by amplification of a family of polymorphic pseudokinases. Proc. Natl. Acad. Sci. USA.

[28] Reese M.L., Zeiner G.M., Saeij J.P.J., Boothroyd J.C., Boyle J.P. (2011). Polymorphic family of injected pseudokinases is paramount in Toxoplasma virulence. Proc. Natl. Acad. Sci. USA.

[29] Etheridge R.D., Alaganan A., Tang K., Lou H.J., Turk B.E., Sibley L.D. (2014). The Toxoplasma pseudokinase ROP5 forms complexes with ROP18 and ROP17 kinases that synergize to control acute virulence in mice. Cell Host Microbe.

[30] Fentress S.J., Behnke M.S., Dunay I.R., Mashayekhi M., Rommereim L.M., Fox B.A., Bzik D.J., Taylor G.A., Turk B.E., Lichti C.F. (2010). Phosphorylation of immunity-related GTPases by a *Toxoplasma gondii*-secreted kinase promotes macrophage survival and virulence. Cell Host Microbe.

[31] Alaganan A., Fentress S.J., Tang K., Wang Q., Sibley L.D. (2014). Toxoplasma GRA7 effector increases turnover of immunity-related GTPases and contributes to acute virulence in the mouse. Proc. Natl. Acad. Sci. USA.

[32] Nyonda M.A., Hammoudi P.-M., Ye S., Maire J., Marq J.-B., Yamamoto M., Soldati-Favre D. (2021). *Toxoplasma gondii* GRA60 is an effector protein that modulates host cell autonomous immunity and contributes to virulence. Cell. Microbiol..

[33] Kim E.W., Nadipuram S.M., Tetlow A.L., Barshop W.D., Liu P.T., Wohlschlegel J.A., Bradley P.J. (2016). The Rhoptry Pseudokinase ROP54 Modulates *Toxoplasma gondii* Virulence and Host GBP2 Loading. mSphere.

[34] Fox B.A., Guevara R.B., Rommereim L.M., Falla A., Bellini V., Pètre G., Rak C., Cantillana V., Dubremetz J.-F., Cesbron-Delauw M.-F. (2019). *Toxoplasma gondii* Parasitophorous Vacuole Membrane-Associated Dense Granule Proteins Orchestrate Chronic Infection and GRA12 Underpins Resistance to Host Gamma Interferon. mBio.

[35] Rosowski E.E., Lu D., Julien L., Rodda L., Gaiser R.A., Jensen K.D.C., Saeij J.P.J. (2011). Strain-specific activation of the NF-κB pathway by GRA15, a novel *Toxoplasma gondii* dense granule protein. J. Exp. Med..

[36] Wang P., Li S., Zhao Y., Zhang B., Li Y., Liu S., Du H., Cao L., Ou M., Ye X. (2019). The GRA15 protein from *Toxoplasma gondii* enhances host defense responses by activating the interferon stimulator STING. J. Biol. Chem..

[37] Sangaré L.O., Yang N., Konstantinou E.K., Lu D., Mukhopadhyay D., Young L.H., Saeij J.P.J. (2019). Toxoplasma GRA15 Activates the NF-κB Pathway through Interactions with TNF Receptor-Associated Factors. mBio.

[38] Mukhopadhyay D., Sangaré L.O., Braun L., Hakimi M.-A., Saeij J.P. (2020). Toxoplasma GRA15 limits parasite growth in IFNγ-activated fibroblasts through TRAF ubiquitin ligases. EMBO J..

[39] Xu Y., Wang X., Liu J., Fu Y., Xu J., Liu Q. (2018). *Toxoplasma gondii* rhoptry protein38 (TgROP38) affects parasite invasion, egress, and induces IL-18 secretion during early infection. Acta Biochim. Biophys. Sin..

[40] Fox B.A., Rommereim L.M., Guevara R.B., Falla A., Hortua Triana M.A., Sun Y., Bzik D.J. (2016). The *Toxoplasma gondii* Rhoptry Kinome Is Essential for Chronic Infection. mBio.

[41] Melo M.B., Nguyen Q.P., Cordeiro C., Hassan M.A., Yang N., McKell R., Rosowski E.E., Julien L., Butty V., Dardé M.-L. (2013). Transcriptional analysis of murine macrophages infected with different Toxoplasma strains identifies novel regulation of host signaling pathways. PLoS Pathog..

[42] Peixoto L., Chen F., Harb O.S., Davis P.H., Beiting D.P., Brownback C.S., Ouloguem D., Roos D.S. (2010). Integrative genomic approaches highlight a family of parasite-specific kinases that regulate host responses. Cell Host Microbe.

[43] Ma J.S., Sasai M., Ohshima J., Lee Y., Bando H., Takeda K., Yamamoto M. (2014). Selective and strain-specific NFAT4 activation by the *Toxoplasma gondii* polymorphic dense granule protein GRA6. J. Exp. Med..

[44] Shastri A.J., Marino N.D., Franco M., Lodoen M.B., Boothroyd J.C. (2014). GRA25 is a novel virulence factor of *Toxoplasma gondii* and influences the host immune response. Infect. Immun..

[45] He H., Brenier-Pinchart M.-P., Braun L., Kraut A., Touquet B., Couté Y., Tardieux I., Hakimi M.-A., Bougdour A. (2018). Characterization of a Toxoplasma effector uncovers an alternative GSK3/β-catenin-regulatory pathway of inflammation. eLife.

[46] Tomita T., Mukhopadhyay D., Han B., Yakubu R., Tu V., Mayoral J., Sugi T., Ma Y., Saeij J.P.J., Weiss L.M. (2021). *Toxoplasma gondii* Matrix Antigen 1 Is a Secreted Immunomodulatory Effector. mBio.

[47] Saeij J.P.J., Boyle J.P., Coller S., Taylor S., Sibley L.D., Brooke-Powell E.T., Ajioka J.W., Boothroyd J.C. (2006). Polymorphic secreted kinases are key virulence factors in toxoplasmosis. Science.

[48] Jensen K.D.C., Hu K., Whitmarsh R.J., Hassan M.A., Julien L., Lu D., Chen L., Hunter C.A., Saeij J.P.J. (2013). *Toxoplasma gondii* rhoptry 16 kinase promotes host resistance to oral infection and intestinal inflammation only in the context of the dense granule protein GRA15. Infect. Immun..

[49] Rudzki E.N., Ander S.E., Coombs R.S., Alrubaye H.I., Cabo L.F., Blank M.L., Gutierrez-Melo N., Dubey J.P., Coyne C.B., Boyle J.P. (2020). *Toxoplasma gondii* GRA28 is required for specific induction of the regulatory chemokine CCL22 in human and mouse cells. bioRxiv.

[50] Braun L., Brenier-Pinchart M.-P., Yogavel M., Curt-Varesano A., Curt-Bertini R.-L., Hussain T., Kieffer-Jaquinod S., Coute Y., Pelloux H., Tardieux I. (2013). A Toxoplasma dense granule protein, GRA24, modulates the early immune response to infection by promoting a direct and sustained host p38 MAPK activation. J. Exp. Med..

[51] Bougdour A., Durandau E., Brenier-Pinchart M.-P., Ortet P., Barakat M., Kieffer S., Curt-Varesano A., Curt-Bertini R.-L., Bastien O., Coute Y. (2013). Host cell subversion by Toxoplasma GRA16, an exported dense granule protein that targets the host cell nucleus and alters gene expression. Cell Host Microbe.

[52] Braun L., Brenier-Pinchart M.-P., Hammoudi P.-M., Cannella D., Kieffer-Jaquinod S., Vollaire J., Josserand V., Touquet B., Couté Y., Tardieux I. (2019). The Toxoplasma effector TEEGR promotes parasite persistence by modulating NF-κB signalling via EZH2. Nat. Microbiol..

[53] Panas M.W., Naor A., Cygan A.M., Boothroyd J.C. (2019). Toxoplasma Controls Host Cyclin E Expression through the Use of a Novel MYR1-Dependent Effector Protein, HCE1. mBio.

[54] Gay G., Braun L., Brenier-Pinchart M.-P., Vollaire J., Josserand V., Bertini R.-L., Varesano A., Touquet B., De Bock P.-J., Coute Y. (2016). *Toxoplasma gondii* TgIST co-opts host chromatin repressors dampening STAT1-dependent gene regulation and IFN-γ–mediated host defenses. J. Exp. Med..

[55] Olias P., Etheridge R.D., Zhang Y., Holtzman M.J., Sibley L.D. (2016). Toxoplasma Effector Recruits the Mi-2/NuRD Complex to Repress STAT1 Transcription and Block IFN-γ-Dependent Gene Expression. Cell Host Microbe.

[56] Rosenberg A., David Sibley L. (2021). *Toxoplasma gondii* secreted effectors co-opt host repressor complexes to inhibit necroptosis. Cell Host Microbe.

[57] Martens S., Howard J. (2006). The interferon-inducible GTPases. Annu. Rev. Cell Dev. Biol..

[58] Khaminets A., Hunn J.P., Könen-Waisman S., Zhao Y.O., Preukschat D., Coers J., Boyle J.P., Ong Y.-C., Boothroyd J.C., Reichmann G. (2010). Coordinated loading of IRG resistance GTPases on to the *Toxoplasma gondii* parasitophorous vacuole. Cell. Microbiol..

[59] Taylor S., Barragan A., Su C., Fux B., Fentress S.J., Tang K., Beatty W.L., Hajj H.E., Jerome M., Behnke M.S. (2006). A secreted serine-threonine kinase determines virulence in the eukaryotic pathogen *Toxoplasma gondii*. Science.

[60] Fleckenstein M.C., Reese M.L., Könen-Waisman S., Boothroyd J.C., Howard J.C., Steinfeldt T. (2012). A *Toxoplasma gondii* Pseudokinase Inhibits Host IRG Resistance Proteins. PLoS Biol..

[61] Murillo-León M., Müller U.B., Zimmermann I., Singh S., Widdershooven P., Campos C., Alvarez C., Könen-Waisman S., Lukes N., Ruzsics Z. (2019). Molecular mechanism for the control of virulent *Toxoplasma gondii* infections in wild-derived mice. Nat. Commun..

[62] Hermanns T., Müller U.B., Könen-Waisman S., Howard J.C., Steinfeldt T. (2015). The *Toxoplasma gondii* rhoptry protein ROP18 is an Irga6-specific kinase and regulated by the dense granule protein GRA7. Cell. Microbiol..

[63] Michelin A., Bittame A., Bordat Y., Travier L., Mercier C., Dubremetz J.-F., Lebrun M. (2009). GRA12, a Toxoplasma dense granule protein associated with the intravacuolar membranous nanotubular network. Int. J. Parasitol..

[64] Wang J.-L., Bai M.-J., Elsheikha H.M., Liang Q.-L., Li T.-T., Cao X.-Z., Zhu X.-Q. (2020). Novel roles of dense granule protein 12 (GRA12) in *Toxoplasma gondii* infection. FASEB J..

[65] Shi J.-H., Sun S.-C. (2018). Tumor Necrosis Factor Receptor-Associated Factor Regulation of Nuclear Factor κB and Mitogen-Activated Protein Kinase Pathways. Front. Immunol..

[66] Pandori W.J., Lima T.S., Mallya S., Kao T.H., Gov L., Lodoen M.B. (2019). *Toxoplasma gondii* activates a Syk-CARD9-NF-κB signaling axis and gasdermin D-independent release of IL-1β during infection of primary human monocytes. PLoS Pathog..

[67] Morgado P., Sudarshana D.M., Gov L., Harker K.S., Lam T., Casali P., Boyle J.P., Lodoen M.B. (2014). Type II *Toxoplasma gondii* induction of CD40 on infected macrophages enhances interleukin-12 responses. Infect. Immun..

[68] Bando H., Lee Y., Sakaguchi N., Pradipta A., Ma J.S., Tanaka S., Cai Y., Liu J., Shen J., Nishikawa Y. (2018). Inducible Nitric Oxide Synthase Is a Key Host Factor for Toxoplasma GRA15-Dependent Disruption of the Gamma Interferon-Induced Antiparasitic Human Response. mBio.

[69] Clough B., Wright J.D., Pereira P.M., Hirst E.M., Johnston A.C., Henriques R., Frickel E.-M. (2016). K63-Linked Ubiquitination Targets *Toxoplasma gondii* for Endo-lysosomal Destruction in IFNγ-Stimulated Human Cells. PLoS Pathog..

[70] Mercier C., Dubremetz J.-F., Rauscher B., Lecordier L., Sibley L.D., Cesbron-Delauw M.-F. (2002). Biogenesis of nanotubular network in Toxoplasma parasitophorous vacuole induced by parasite proteins. Mol. Biol. Cell.

[71] Tu V., Tomita T., Sugi T., Mayoral J., Han B., Yakubu R.R., Williams T., Horta A., Ma Y., Weiss L.M. (2020). The *Toxoplasma gondii* Cyst Wall Interactome. mBio.

[72] Dunay I.R., Damatta R.A., Fux B., Presti R., Greco S., Colonna M., Sibley L.D. (2008). Gr1(+) inflammatory monocytes are required for mucosal resistance to the pathogen *Toxoplasma gondii*. Immunity.

[73] Robben P.M., LaRegina M., Kuziel W.A., Sibley L.D. (2005). Recruitment of Gr-1+ monocytes is essential for control of acute toxoplasmosis. J. Exp. Med..

[74] Goldszmid R.S., Coppens I., Lev A., Caspar P., Mellman I., Sher A. (2009). Host ER-parasitophorous vacuole interaction provides a route of entry for antigen cross-presentation in *Toxoplasma gondii*-infected dendritic cells. J. Exp. Med..

[75] Yamamoto M., Ma J.S., Mueller C., Kamiyama N., Saiga H., Kubo E., Kimura T., Okamoto T., Okuyama M., Kayama H. (2011). ATF6{beta} is a host cellular target of the *Toxoplasma gondii* virulence factor ROP18. J. Exp. Med..

[76] An R., Tang Y., Chen L., Cai H., Lai D.-H., Liu K., Wan L., Gong L., Yu L., Luo Q. (2018). Encephalitis is mediated by ROP18 of *Toxoplasma gondii*, a severe pathogen in AIDS patients. Proc. Natl. Acad. Sci. USA.

[77] Kahali S., Sarcar B., Prabhu A., Seto E., Chinnaiyan P. (2012). Class I histone deacetylases localize to the endoplasmic reticulum and modulate the unfolded protein response. FASEB J..

[78] Bertolotti A., Zhang Y., Hendershot L.M., Harding H.P., Ron D. (2000). Dynamic interaction of BiP and ER stress transducers in the unfolded-protein response. Nat. Cell Biol..

[79] Steinhart Z., Angers S. (2018). Wnt signaling in development and tissue homeostasis. Development.

[80] Romagnani S. (2002). Cytokines and chemoattractants in allergic inflammation. Mol. Immunol..

[81] Rapp M., Wintergerst M.W.M., Kunz W.G., Vetter V.K., Knott M.M.L., Lisowski D., Haubner S., Moder S., Thaler R., Eiber S. (2019). CCL22 controls immunity by promoting regulatory T cell communication with dendritic cells in lymph nodes. J. Exp. Med..

[82] Parmley S.F., Yang S., Harth G., Sibley L.D., Sucharczuk A., Remington J.S. (1994). Molecular characterization of a 65-kilodalton *Toxoplasma gondii* antigen expressed abundantly in the matrix of tissue cysts. Mol. Biochem. Parasitol..

[83] Ewald S.E., Chavarria-Smith J., Boothroyd J.C. (2014). NLRP1 is an inflammasome sensor for *Toxoplasma gondii*. Infect. Immun..

[84] Gorfu G., Cirelli K.M., Melo M.B., Mayer-Barber K., Crown D., Koller B.H., Masters S., Sher A., Leppla S.H., Moayeri M. (2014). Dual role for inflammasome sensors NLRP1 and NLRP3 in murine resistance to *Toxoplasma gondii*. mBio.

[85] Kelley N., Jeltema D., Duan Y., He Y. (2019). The NLRP3 Inflammasome: An Overview of Mechanisms of Activation and Regulation. Int. J. Mol. Sci..

[86] Saeij J.P.J., Coller S., Boyle J.P., Jerome M.E., White M.W., Boothroyd J.C. (2007). Toxoplasma co-opts host gene expression by injection of a polymorphic kinase homologue. Nature.

[87] Ong Y.-C., Reese M.L., Boothroyd J.C. (2010). Toxoplasma rhoptry protein 16 (ROP16) subverts host function by direct tyrosine phosphorylation of STAT6. J. Biol. Chem..

[88] Yamamoto M., Standley D.M., Takashima S., Saiga H., Okuyama M., Kayama H., Kubo E., Ito H., Takaura M., Matsuda T. (2009). A single polymorphic amino acid on *Toxoplasma gondii* kinase ROP16 determines the direct and strain-specific activation of Stat3. J. Exp. Med..

[89] Kaplan M.H., Schindler U., Smiley S.T., Grusby M.J. (1996). Stat6 is required for mediating responses to IL-4 and for development of Th2 cells. Immunity.

[90] Jensen K.D.C., Wang Y., Wojno E.D.T., Shastri A.J., Hu K., Cornel L., Boedec E., Ong Y.-C., Chien Y.-H., Hunter C.A. (2011). Toxoplasma polymorphic effectors determine macrophage polarization and intestinal inflammation. Cell Host Microbe.

[91] Nadipuram S.M., Kim E.W., Vashisht A.A., Lin A.H., Bell H.N., Coppens I., Wohlschlegel J.A., Bradley P.J. (2016). In Vivo Biotinylation of the Toxoplasma Parasitophorous Vacuole Reveals Novel Dense Granule Proteins Important for Parasite Growth and Pathogenesis. mBio.

[92] Franco M., Panas M.W., Marino N.D., Lee M.C.W., Buchholz K.R., Kelly F.D., Bednarski J.J., Sleckman B.P., Pourmand N., Boothroyd J.C. (2016). A novel secreted protein, MYR1, is central to Toxoplasma’s manipulation of host cells. mBio.

[93] Pellegrini E., Palencia A., Braun L., Kapp U., Bougdour A., Belrhali H., Bowler M.W., Hakimi M.-A. (2017). Structural Basis for the Subversion of MAP Kinase Signaling by an Intrinsically Disordered Parasite Secreted Agonist. Structure.

[94] Mercer H.L., Snyder L.M., Doherty C.M., Fox B.A., Bzik D.J., Denkers E.Y. (2020). *Toxoplasma gondii* dense granule protein GRA24 drives MyD88-independent p38 MAPK activation, IL-12 production and induction of protective immunity. PLoS Pathog..

[95] Mukhopadhyay D., Arranz-Solís D., Saeij J.P.J. (2020). Toxoplasma GRA15 and GRA24 are important activators of the host innate immune response in the absence of TLR11. PLoS Pathog..

[96] Li M., Chen D., Shiloh A., Luo J., Nikolaev A.Y., Qin J., Gu W. (2002). Deubiquitination of p53 by HAUSP is an important pathway for p53 stabilization. Nature.

[97] Kim S.-G., Seo S.-H., Shin J.-H., Yang J.-P., Lee S.H., Shin E.-H. (2019). Increase in the nuclear localization of PTEN by the Toxoplasma GRA16 protein and subsequent induction of p53-dependent apoptosis and anticancer effect. J. Cell. Mol. Med..

[98] Seo S.-H., Kim S.-G., Shin J.-H., Ham D.-W., Shin E.-H. (2020). Toxoplasma GRA16 Inhibits NF-κB Activation through PP2A-B55 Upregulation in Non-Small-Cell Lung Carcinoma Cells. Int. J. Mol. Sci..

[99] Panas M.W., Boothroyd J.C. (2020). Toxoplasma Uses GRA16 To Upregulate Host c-Myc. mSphere.

[100] Kent L.N., Leone G. (2019). The broken cycle: E2F dysfunction in cancer. Nat. Rev. Cancer.

[101] Kouznetsova V.L., Tchekanov A., Li X., Yan X., Tsigelny I.F. (2019). Polycomb repressive 2 complex-Molecular mechanisms of function. Protein Sci..

[102] Matta S.K., Olias P., Huang Z., Wang Q., Park E., Yokoyama W.M., Sibley L.D. (2019). *Toxoplasma gondii* effector TgIST blocks type I interferon signaling to promote infection. Proc. Natl. Acad. Sci. USA.

[103] Yap G.S., Sher A. (1999). Effector cells of both nonhemopoietic and hemopoietic origin are required for interferon (IFN)-gamma- and tumor necrosis factor (TNF)-alpha-dependent host resistance to the intracellular pathogen, *Toxoplasma gondii*. J. Exp. Med..

[104] Han S.-J., Melichar H.J., Coombes J.L., Chan S.W., Koshy A.A., Boothroyd J.C., Barton G.M., Robey E.A. (2014). Internalization and TLR-dependent type I interferon production by monocytes in response to *Toxoplasma gondii*. Immunol. Cell Biol..

[105] Torchy M.P., Hamiche A., Klaholz B.P. (2015). Structure and function insights into the NuRD chromatin remodeling complex. Cell. Mol. Life Sci..

[106] Mayoral J., Shamamian P., Weiss L.M. (2020). In Vitro Characterization of Protein Effector Export in the Bradyzoite Stage of *Toxoplasma gondii*. mBio.

[107] Krishnamurthy S., Saeij J.P.J. (2018). Toxoplasma Does Not Secrete the GRA16 and GRA24 Effectors Beyond the Parasitophorous Vacuole Membrane of Tissue Cysts. Front. Cell. Infect. Microbiol..

[108] Watson P.J., Fairall L., Schwabe J.W.R. (2012). Nuclear hormone receptor co-repressors: Structure and function. Mol. Cell. Endocrinol..

[109] Galluzzi L., Kepp O., Chan F.K.-M., Kroemer G. (2017). Necroptosis: Mechanisms and Relevance to Disease. Annu. Rev. Pathol..

[110] Rastogi S., Cygan A.M., Boothroyd J.C. (2019). Translocation of effector proteins into host cells by *Toxoplasma gondii*. Curr. Opin. Microbiol..

[111] Wang Y., Sangaré L.O., Paredes-Santos T.C., Hassan M.A., Krishnamurthy S., Furuta A.M., Markus B.M., Lourido S., Saeij J.P.J. (2020). Genome-wide screens identify *Toxoplasma gondii* determinants of parasite fitness in IFNγ-activated murine macrophages. Nat. Commun..

[112] Gold D.A., Kaplan A.D., Lis A., Bett G.C.L., Rosowski E.E., Cirelli K.M., Bougdour A., Sidik S.M., Beck J.R., Lourido S. (2015). The Toxoplasma Dense Granule Proteins GRA17 and GRA23 Mediate the Movement of Small Molecules between the Host and the Parasitophorous Vacuole. Cell Host Microbe.

[113] Marino N.D., Panas M.W., Franco M., Theisen T.C., Naor A., Rastogi S., Buchholz K.R., Lorenzi H.A., Boothroyd J.C. (2018). Identification of a novel protein complex essential for effector translocation across the parasitophorous vacuole membrane of *Toxoplasma gondii*. PLoS Pathog..

[114] Panas M.W., Ferrel A., Naor A., Tenborg E., Lorenzi H.A., Boothroyd J.C. (2019). Translocation of Dense Granule Effectors across the Parasitophorous Vacuole Membrane in Toxoplasma-Infected Cells Requires the Activity of ROP17, a Rhoptry Protein Kinase. mSphere.

[115] Tu V., Mayoral J., Sugi T., Tomita T., Han B., Ma Y.F., Weissa L.M. (2019). Enrichment and proteomic characterization of the cyst wall from in vitro *Toxoplasma gondii* cysts. MBio.

[116] Mayoral J., Tomita T., Tu V., Aguilan J.T., Sidoli S., Weiss L.M. (2020). *Toxoplasma gondii* PPM3C, a secreted protein phosphatase, affects parasitophorous vacuole effector export. PLoS Pathog..

[117] Tomita T., Bzik D.J., Ma Y.F., Fox B.A., Markillie L.M., Taylor R.C., Kim K., Weiss L.M. (2013). The *Toxoplasma gondii* Cyst Wall Protein CST1 Is Critical for Cyst Wall Integrity and Promotes Bradyzoite Persistence. PLoS Pathog..

[118] Rastogi S., Xue Y., Quake S.R., Boothroyd J.C. (2020). Differential Impacts on Host Transcription by ROP and GRA Effectors from the Intracellular Parasite *Toxoplasma gondii*. mBio.

[119] Xue Y., Theisen T.C., Rastogi S., Ferrel A., Quake S.R., Boothroyd J.C. (2020). A single-parasite transcriptional atlas of *Toxoplasma gondii* reveals novel control of antigen expression. eLife.

[120] Patir A., Gossner A., Ramachandran P., Alves J., Freeman T.C., Henderson N.C., Watson M., Hassan M.A. (2020). Single-cell RNA-seq reveals CD16- monocytes as key regulators of human monocyte transcriptional response to Toxoplasma. Sci. Rep..

[121] Coffey M.J., Sleebs B.E., Uboldi A.D., Garnham A.L., Franco M., Marino N.D., Panas M.W., Ferguson D.J., Enciso M., O’Neill M.T. (2015). An aspartyl protease defines a novel pathway for export of Toxoplasma proteins into the host cell. eLife.

[122] Hammoudi P.-M., Jacot D., Mueller C., Di Cristina M., Dogga S.K., Marq J.-B., Romano J., Tosetti N., Dubrot J., Emre Y. (2015). Fundamental Roles of the Golgi-Associated Toxoplasma Aspartyl Protease, ASP5, at the Host-Parasite Interface. PLoS Pathog..

